# Snake fungal disease alters skin bacterial and fungal diversity in an endangered rattlesnake

**DOI:** 10.1038/s41598-018-30709-x

**Published:** 2018-08-14

**Authors:** Matthew C. Allender, Sarah Baker, Megan Britton, Angela D. Kent

**Affiliations:** 1 Wildlife Epidemiology Lab, Department of Veterinary Clinical Medicine, College of Veterinary Medicine, University of Illinois Urbana-Champaign, Urbana, IL USA; 2 Illinois Natural History Survey, Prairie Research Institute, University of Illinois Urbana-Champaign, Champaign, IL USA; 3 Department of Natural Resources and Environmental Sciences, University of Illinois Urbana-Champaign, Champaign, IL USA

## Abstract

Snake Fungal Disease (SFD), caused by *Ophidiomyces ophiodiicola*, is the most recently described fungal disease afflicting wildlife populations across North America and Europe. It has been proposed as a significant conservation threat yielding high mortality and yet much its ecology is unknown. We collected 144 skin swabs from Eastern Massasaugas (*Sistrurus catenatus*) in 2015 and 2016 to determine document ongoing prevalence and assess differences in microbial assemblages between positive and negative individuals. Alpha diversity of fungi was reduced in SFD positive animals, while beta diversity identified distinct assemblages of microbes between SFD–positive and –negative samples. *Ophidiomyces* was present on the skin of affected animals, even on body sites distant to lesions indicating that the microbiome on entire surface of the skin is altered. *Ophidiomyces* was not detected in any non-SFD snake. There were smaller, but significant, influences of year sampled. Bacterial genera *Janthinobacterium* and *Serratia* were significantly increased in SFD snakes, while *Xylanimicrobium*, *Cellulosimicrobium*, and *Rhodococcus* were the only bacterial taxa significantly reduced. The relative abundance of fungi within the orders Pleosporales and Canopdiales was reduced in SFD-positive samples, though *Pyrenochaetopsis pratorum* was the only species found to differ significantly. This is the first study to determine the impact that this fungal pathogen has on the skin microbiome.

## Introduction

Global fungal pathogens are increasingly associated with free-ranging wildlife epidemics^1^. Chytridiomycosis, caused by *Batrachochytrium dendrobatidis (Bd)* threatens amphibian populations worldwide^2^, *Pseudogymnoascus destructans*, has decimated North American bat populations^3^, and the emergence of *Ophidiomyces ophiodiicola* (Snake Fungal Disease; SFD) in 2000 is alarming because of its broad geographic and taxonomic distributions in the US, Canada, and Europe^4,5^. *Ophidiomyces* has been documented from the skin of Eastern Massasaugas (*Sistrurus catenatus*)^6,7^, Timber Rattlesnakes (*Crotalus horridus*)^8^, pigmy rattlesnakes (*Sistrurus miliarius barbouri*)^9^, and several colubrid snakes^10–12^. Yet host and environmental factors that lead to disease are largely unknown. Here we investigate the skin microbiome of a population of snakes that is consistently observed with SFD to determine its role in contributing to disease susceptibility or disease outcome, or the response of the microbiome to SFD.

The Eastern Massasauga is listed as threatened under the federal endangered species act (USFWS 2016), and in Illinois only one extant population remains. In the eastern US, the Eastern Massasauga (*Sistrurus catenatus*) is specifically vulnerable to disease due to *Ophidiomyces* infections^6^. The known fates of individuals detected with *Ophidiomyces* have shown a greater than 90% mortality (unpub data). To determine the nature of this species’ apparent sensitivity, a comprehensive health assessment program was established that investigated hematology, plasma biochemistries, heavy metal analysis, protein electrophoretograms, and disease epidemiology before and after the emergence of this pathogen in Illinois^4,13–15^. However, there have been very few changes in these health parameters that are associated with SFD.

It is becoming increasingly apparent that environmental microbial communities greatly impact animal health^16,17^, and should be included in health assessment of snakes. In humans, a healthy and diverse skin microbiome is linked to a reduction in infectious and non-infectious skin diseases^18^. The normal microbiota can resist disease occurrence (pathogen-resistance hypothesis) and disruption to it has led to increased occurrence of pathogens in people^19,20^ and bumblebees^21^. Conversely, pathogen-initiated disruption of normal microbiota (pathogen-induced hypothesis) is documented in both humans^22^ and frogs^23,24^. The mechanism driving SFD is completely unknown. This information gap impedes conservation and treatment plans aimed at reducing the impact of this disease in snakes, including Eastern Massasaugas.

Our objectives for this study were to assess the microbial communities associated with skin of Eastern Massasaugas from a population with a persistent occurrence of SFD. Our specific hypotheses were: 1) The microbial composition on the skin of Eastern Massasaugas will be altered in individuals with SFD, 2) Microbial composition will not differ based on demographic factors, such as sex, location, and year sampled, and 3) The presence of co-pathogens may be identified in individuals with *Ophidiomyces*.

## Results

### General Survey and Ophidiomyces Detection

We collected swabs from 44 Eastern Massasaugas in 2015 (66 swabs) and 52 individuals (61 swabs) in 2016. Six animals were tested in both 2015 and 2016. We sampled the most individuals at Eldon Hazlet State Park (EHSP; n = 55), followed by South Shore State Park (SSSP; N = 30), Field 3 (N = 23), and Dam East (DE; N = 2) all near Carlyle Lake, IL. We sampled 57 males, 52 females, and 1 snake with unknown sex. Thirty-six of the swabs we collected were from lesions, while 96 swabs were from apparently healthy skin from the face.

We identified 31 samples from 16 animals with *Ophidiomyces*, for an overall prevalence of 17.8% (95% CI: 10.5–27.3%). In these animals, the presence of dermatitis was significantly associated with presence of *Ophidiomyces* (P < 0.001), as 93.8% (N = 15) of snakes detected with *Ophidiomyces* DNA had lesions, while only one (6.2%) snake with *Ophidiomyces* had no lesions. Of the six individuals sampled in both years, four were negative in both years, one was negative in 2015, but positive in 2016, and one that was positive in both years. Most samples were collected from the face (N = 99), followed by the body (N = 23), and tail (N = 5). The results of body site detection reflected the study design in which the face was sampled in the majority of individuals, while body and tail were only sampled in animals with lesions. Thus, ten (10% of facial swabs), 18 (78% of body swabs), and 5 (100% of tail swabs) samples were positive from the face, body, and tail, respectively.

SFD is a clinical syndrome associated with *Ophidiomyces*, thus we assigned SFD-positive status to animals with detectable *Ophidiomyces* via qPCR and the presence of gross pathological signs consistent with SFD (skin swelling, crusts, papules, skin ulcers). Samples were then assigned to one of three categories for microbiome analysis based on if the sample was detected with *Ophidiomyces* in an SFD individual (sample positive-animal positive), not detected with *Ophidiomyces*, but the animal was determined to be SFD positive based on other positive body sites (sample negative-animal positive), or if the sample was not detected with *Ophidiomyces* and the individual was SFD negative (sample negative-animal negative). Of the sixteen individuals that were detected with *Ophidiomyces* and were swabbed at multiple body sites, there was variable detection of *Ophidiomyces* from these swabs (50%-100% of swabs per individual). Fungal copy numbers in the 31 positive swabs ranged from 0.5–4174 copies/ng total DNA.

### Bacterial and Fungal Community Structure

#### Alpha diversity

**Figure 1:**
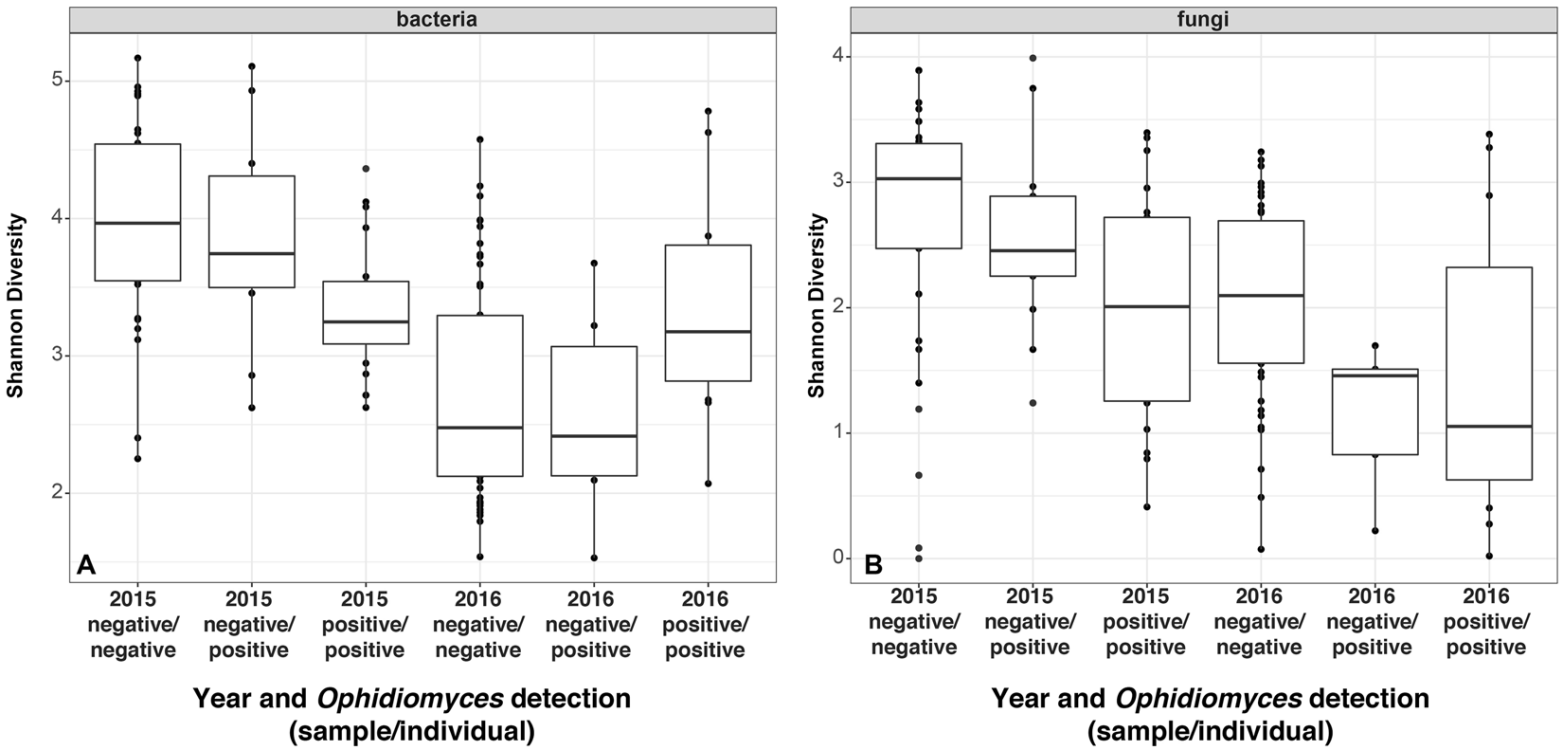
Alpha diversity (Shannon) of bacteria (**A**) and fungi (**B**) on the skin of Eastern Massasauga rattlesnakes (*Sistrurus catenatus*) in each year. Samples were classified as positive or negative based on qPCR targeting *Ophidiomyces ophiodiicola*.

Significantly reduced Shannon species diversity was observed for both bacteria and fungi for samples collected in 2016 (P << 0.001) (Fig. 1). Alpha diversity for fungi was also reduced in samples that were positive for *Ophidiomyces* (P = 0.029 for fungi), and reduced diversity of bacterial communities from *Ophidiomyces*-positive samples was also observed in 2015 (p = 0.002).

#### Beta diversity

**Figure 2:**
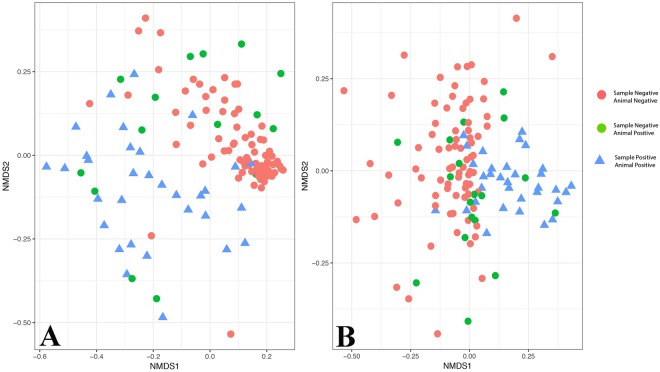
Nonmetric multidimensional scaling analysis of bacterial (**A**) and fungal assemblages (**B**) based on Illumina MiSeq sequencing of bacterial 16S rRNA genes or fungal ITS2, respectively. Each point represents a sample, and the distance between samples represents the Bray-Curtis dissimilarity values calculated among samples, coded by *Ophidiomyces* detection in the sample and SFD status of each individual snake.

For both fungal and bacterial communities, distinct assemblages of microbes were observed between SFD–positive and –negative samples (Fig. 2A and B). Distinctions among microbial assemblages are supported by analysis of similarity (ANOSIM): bacteria: ANOSIM R = 0.39, P < 0.001; fungi: ANOSIM R = 0.16, P = 0.004. ANOSIM R varies between 0 and 1, with 0 representing no differences among classes, and 1 representing complete difference in microbial assemblage. This analysis supports modest but sigificant differences among both bacterial and fungal assemblages based on SFD status.

Other classifications were considered as potential drivers influencing the microbial assemblages. The effect size and statistical significance of sample classifications (SFD status of samples or individuals, year, field site, and sex) on bacterial and fungal community composition were determined using Permutational Multivariate Analysis of Variance (PERMANOVA).

SFD status explains about 7.1% of bacterial community variance (Table 1), and 5.4% of variance in the fungal community (Table 2). The importance of other factors was less pronounced (Tables 1 and 2, Figs S1–S3). Year was a somewhat important factor for bacteria (R^2^ = 0.090), but less important in the fungal communities (R^2^ = 0.042) (Fig. S1). In particular, it was surprising that field site had little effect on microbial community assemblages. This is also supported by NMDS ordinations (Fig. S2), and PERMANOVA: bacteria: R^2^ = 0.032, p < 0.001; fungi: R^2^ = 0.039, p < 0.001.

**Table 1:** Bacterial PERMANOVA model summary for microbial diversity from skin swabs of eastern massasauga rattlesnakes (*Sistrurus catenatus*).

Parameter	Df	Sums of Squares	R^2^	p-value
SFD	1	2.381	0.07075	0.001
Location	2	1.079	0.03206	0.004
Year	1	3.023	0.0898	0.001
Sex	2	0.58	0.01724	0.246
Body Site	2	2.384	0.07084	0.001
SFD Abundance	1	0.673	0.02	0.001

**Table 2:** Fungal PERMANOVA model summary for microbial diversity from skin swabs of eastern massasauga rattlesnakes (*Sistrurus catenatus*).

Parameter	Df	Sums of Squares	R^2^	p-value
SFD	1	2.654	0.05424	0.001
Location	2	1.93	0.03944	0.001
Year	1	2.062	0.04214	0.001
Sex	2	0.959	0.0196	0.098
Body Site	2	2.377	0.04857	0.001
SFD Abundance	1	0.791	0.01617	0.004
Individual SFD status	1	2.041	0.0417	0.001

#### Taxa summaries

**Figure 3:**
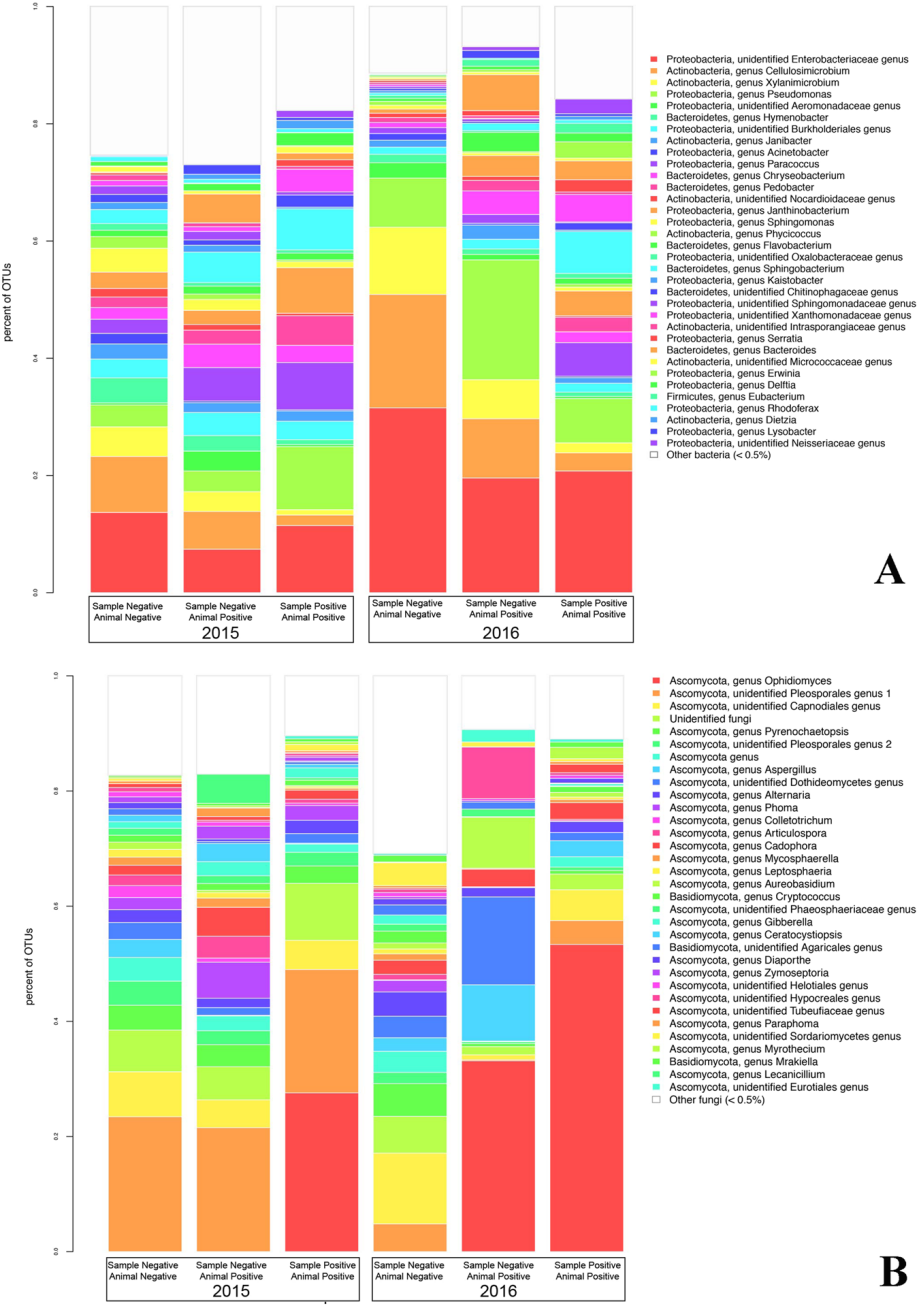
Summary of bacterial (**A**) and fungal (**B**) taxa detected in SFD positive and negative samples and individual, based on Illumina MiSeq sequencing. Classes of samples are displayed separately for each year. Phylogenetic classifications at the genus level are shown, though some genera in this dataset are exclusively comprised of a single species (e.g. *Ophidiomyces ophiodiicola*). Samples were classified as positive or negative based on qPCR targeting *Ophidiomyces ophiodiicola*, and animals were classified as positive based on the presence of clinical signs and detection of *Ophidiomyces* in at least one sample.

**Figure 4:**
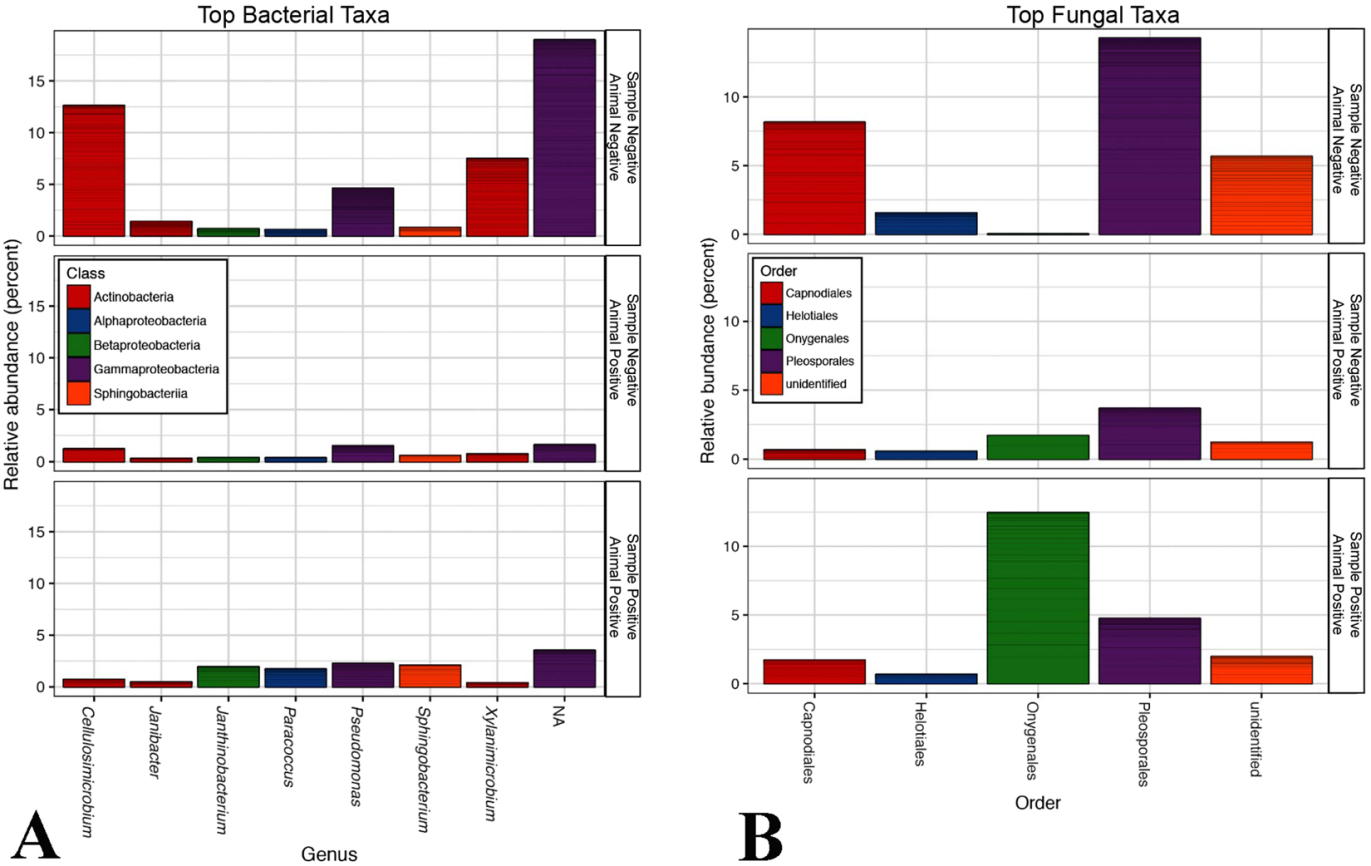
Summary of dominant (top 10) bacterial (**A**) and fungal (**B**) taxa detected with *Ophidiomyces* in samples that were SFD positive (sample positive-animal positive), without *Ophidiomyces* in SFD-positive individuals (sample negative-animal positive), and without *Ophidiomyces* in SFD-negative individuals (sample negative-animal negative).

**Figure 5:**
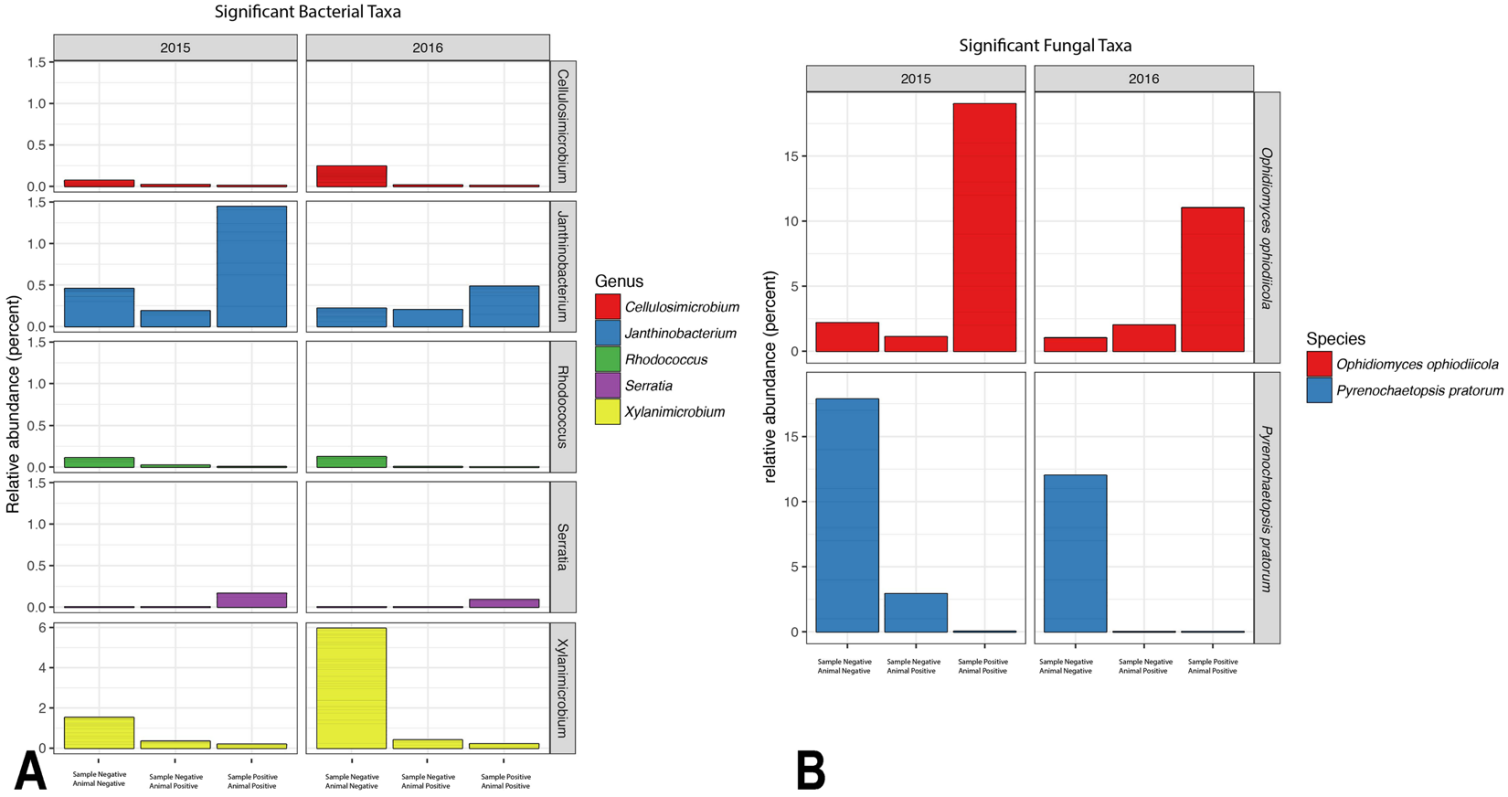
Summary of bacterial (**A**) and fungal (**A**) taxa significantly disrupted with *Ophidiomyces* in samples that were SFD positive (sample positive-animal positive), without *Ophidiomyces* in SFD-positive individuals (sample negative-animal positive), and without *Ophidiomyces* in SFD-negative individuals (sample negative-animal negative).

A total of 7,460,094 reads were generated for fungal ITS2, and 9,038,936 reads were generated for bacterial 16S rRNA genes. A total of 1223 fungal OTUs and 3070 bacterial OTUs were retained for analysis. The relative abundance of bacterial and fungal taxa differed by SFD status (Fig. 3). Dominant bacterial taxa included *Pseudomonas*, and taxa within the Burkholderiales, Actinomycetales, and Enterobacteriaceae (Fig. 4A). Bacteria from the genera *Cellulosimicrobium*, *Pseudomonas*, and *Xylanimicrobium*, and an unidentified Enterobacteriaceae genus were greatly reduced in relative abundance in SFD-positive samples, while Xanthomondales and *Paracoccus* OTUs were increased in relative abundance. OTUs from the genera *Xylanimicrobium, Cellulosimicrobium, Janthinobacterium, Serratia*, and *Rhodococcus* were found to be significantly different (P = 0.01) based on sample SFD classification (Fig. 5A).

The fungal microbiome of SFD-positive samples was dominated, predictably, by *Ophidiomyces ophiodiicola* (the only detected species within the order Onygenales), which was also detected in *Ophidiomyces*-negative samples from individuals with other *Ophidiomyces*-positive samples in 2016 (Fig. 4B). The relative abundance of fungi within the orders Pleosporales and Canopdiales was reduced in SFD-positive samples, though only *Ophidiomyces ophiodiicola* and *Pyrenochaetopsis pratorum* were found to differ significantly (P = 0.05) based on sample SFD classification (Fig. 5B).

## Discussion

A better understanding of host microbial communities will provide insight into mechanisms of pathogen emergence, fluctuations in wellness of individuals, and enhance therapeutic intervention aimed at reducing disease impact. Naturally occurring commensal bacterial and fungal species likely interact and subsequently influence the ability of pathogens to cause disease. We found a disruption in microbial communities of SFD-infected Eastern Massasaugas leading to altered bacterial and fungal diversity in affected snakes (pathogen-driven hypothesis). We feel these findings will have broad relevance to other snake species and habitats. Subsequently, SFD presence in North American snake populations may signify *Ophidiomyces* emerged on the landscape or its virulence has changed to facilitate disruption of the microbiome. If the pathogen-resistance hypothesis predominated, our expectation would be that SFD-negative individuals would have low levels of *Ophidiomyces* detectable by qPCR.

The composition of microbes on the skin of SFD-snakes has not been previously documented, but amphibians with *Bd* have shown higher infection intensity with greater disruption in the microbial community structure^24^. Changes in SFD intensity can similarly affect the microbiome, but unlike *Bd*, *Ophidiomyces* abundance has been shown to be difficult to quantify due to poor detection probabilities^25^ and *Ophidiomyces* abundance was not associated with changes in microbial community structure in our analysis (though *Ophidiomyces* infection was associated with shifts in the microbial community). Furthermore, there are key differences in pathogenesis between *Bd* and SFD that may affect the microbial community structure. In particular, *Bd* infection is restricted to the keratinized epithelium, thus is constantly at the interface of skin microbiome. Whereas, SFD causes deeper invasion of the tissues^5^ and in later stages of the disease may be absent in the epithelium^26^. We found *Ophidiomyces* in a single animal without dermatitis, yet their microbiome aligned with the SFD-positive group. It is possible that this animal was early in the disease process and the pathogen had disrupted the microbiome, but not yet led to clinical signs of dermatitis. Following this animal over time may have changed its classification to SFD-positive.

We found a non-significant difference in SFD qPCR prevalence between years, but there was a significant effect of year on microbial communities. It has been proposed that SFD is observed in Timber rattlesnake populations in years with higher rainfall^8^. It is plausible to consider that environmental conditions lead to changes in microbial communities that confer more or less resistance to SFD infection. This has been observed in lizards that were observed with 34% reduction in gut microbiota diversity with a change in temperature of just 2–3 °C^27^. Thus far, there continues to be a gap in connecting SFD and specific environmental characteristics. The window to sample Eastern Massasaugas in Illinois occurs over an 8–10 week period, and our direct measurements of temperature, rainfall, and humidity have not identified any clear pattern that accounts for the difference we observed in both bacterial and fungal community diversity in 2015 and 2016. It is likely that climate related factors will require long-term data, but that may ignore the effect of microclimates each individual snake experiences. Prospective studies at this site in Massasaugas are critical to characterizing environmental contributions to SFD, specifically changes in the soil microbiome during wet or dry years, or across environmental gradients.

*Ophidiomyces* was the dominant fungal species in all SFD-positive samples. The fungal order Pleosporales was subsequently most reduced, with *Pyrenochaetopsis pratorum* as the only fungal species identified with a significant change (Fig. 4B). Pleosporales are a class of Ascomycete fungi most commonly associated with plants or soils, but recently in cases of human infections^28^. There was intermediate abundance of *Pyrenochaetopsis pratorum* in negative samples from SFD-positive individuals possibly indicating this may be targeted for early detection prior to an SFD outbreak. Challenge experiments have identified *Ophidiomyces* as the causative agent in SFD^25,29^, but several case reports have identified other primary fungi based on culture, including *Fusarium* and *Aspergillus*^6,30,31^. *Ophidiomyces* is a slow growing fungus and in these cases, faster growing saprophytes, such as *Fusarium* and *Aspergillus*, on the skin of snakes obscured the cultured results^32^. We detected species in the orders Hypocreales (contains *Fusarium* sp.) and *Eurotiales* (contains *Aspergillus* sp.) in all positive and negative samples supporting these species as opportunistic invaders. The ability of naturally occurring fungi to inhibit or competitively exclude *Ophidiomyces* needs to be studied *in vitro* and we propose that *Pyrenochaetopsis pratorum* should be evaluated first.

We found bacterial species community structure differs in SFD-positive samples. Specifically, Actinomycetales were reduced in relative abundance in SFD-positive samples, while Xanthomondales were increased in relative abundance. The genera that appear to be enriched in SFD-positive samples include *Sphingobacterium*, *Janthinobacterium*, *Paracoccus*, *Pedobacter*, *Erwinia* (in 2016), and unnamed genera within *Xanthomonadaceae* and *Neisseriaceae*. Though this difference was found to be significant only for the genera *Janthinobacterium* and *Serratia*, while *Xylanimicrobium, Cellulosimicrobium*, and *Rhodococcus* were the only taxa significantly reduced (Fig. 5A). It is interesting to note that *Janthinobacterium* has been identified as an anti-*Bd* agent in salamanders^33^. The most common bacterial genera in our study was *Pseudomonas*, a large group of species that include many pathogens of reptiles, and also fungal biocontrol agents. Certain species of *Pseudomonas* have shown mixed activity against *Bd* in amphibians^34,35^. Recently, 16 species of bacteria were identified with inhibitory activity against *Ophidiomyces*, including *Erwinia* sp^36^. However, the anti-*Ophidiomyces* activity of *Erwinia* determined in that study was not as strong as *Morganella morganii* cultured from Timber rattlesnake skin swabs, which the authors propose as a candidate probiotic species^36^. *Morganella* was not a common bacterial genus identified in our study in positive or negative samples^36^. Future studies should determine if *Morganella* has anti-*Ophidiomyces* activity in Eastern massasaugas and is normally detected in healthy individuals.

Previous reports documented the face as a primary site of SFD infection^4^, our study design called for swabbing the face (but not body or tail) of every animal, thus leading to unequal sampling sizes between body sites. Sample sizes of tail and body were much fewer than face and thus the statistical power may not be sufficient to detect the differences in prevalence between body sites. Our PERMANOVA found a significant, but minimal, difference in bacterial and fungal community structure between body sites, which is likely explained by differences in behavior and habitat use of a snake that cause variability in exposure to environmental microbes among body sites. The ventral surface of the body is commonly in contact with soil, whereas the face is commonly elevated above the surface, thus leading to potential differences in the microbiota. It is possible that the presence of the altered bacterial community structure described above may confer differences in disease patterns in our study, and challenge studies that aim to inoculate different body sites are needed.

Snake fungal disease is causing widespread morbidity and mortalites in North America and describing the microbiome and its potential ability to confer disease resistance or susceptibility will provide opportunities to improve conservation efforts. Furthermore, as disruption of the microbial communities might signifiy deteriorating ecosytem health, it may further aid other populations of wildlife or humans relying on these natural resources. Recent evaluation of anti-*Ophidiomyces* activity by a compound utilized to combat fungal diseases in agriculture has garnered concern because of apparent resistance^37^. The Eastern Massasauga appears to be highly sensitive to SFD, thus determining the impact of *Ophidiomyces* on the skin microbiome in other species is warranted. This study generated findings that characterize the role of the microbiome in SFD which can now be utilized to address this fatal disease in the face of changing environmental and physiologic factors including resistance, pollution, climate change, and therapeutic intervention.

## Methods

### General Methods

During 2015 and 2016, during spring egress, we conducted visual encounter surveys of wild Eastern Massasaugas populations around Carlyle Lake, Illinois: 1) South Shore State Park (SSSP), 2) Eldon Hazlet State Park (EHSP), 3) Field 3, and 4) Dam East Recreational site (DE). All individuals were marked either via PIT-tagging or painting rattle segments. We recorded sex, age class, snout-vent length (SVL, cm), tail length (cm), and mass (g) for all individuals. After processing, we returned snakes to their initial points of capture. We disinfected all sampling and handling equipment following reported protocols^37^. This study was approved by the University of Illinois Institutional Animal Care and Use Committee (Protocol: 14000) all methods were performed in accordance with the relevant guidelines and regulations.

### Field Health Assessment

We assessed all animals for clinical signs consistent with SFD, i.e. generalized dermatitis, skin lesions, facial swelling, or discharge. Presence and location of any of these signs was assigned as present/absent on face, body, or tail, respectively. Using sterile cotton-tipped applicators, all individuals were sampled on their face, and if lesions were present on other body sites they were swabbed with a separate applicator. Due to multiple lesions in some individuals, each individual was swabbed between 1 and 3 times, with individuals not demonstrating clinical signs only swabbed once. Samples were stored in 2 ml Eppendorf tubes, frozen at −20 °C until analysis. DNA extraction and quantitative PCR amplification (qPCR) were performed as previously reported for detection of *Ophidiomyces ophiodiicola*^38^. Briefly, qPCR was performed in triplicate on an ABI 7500 real time thermacycler. Samples were considered positive if all three replicates had a lower cycle threshold (C_t_) value than the lowest detected standard dilution. SFD was classified based on the presence of clinical signs and detection of *Ophidiomyces* using qPCR^25^.

### Bacterial and Fungal Community Structure

We prepared bacterial and fungal amplicons by PCR using universal primers targeting the V4 region of the bacterial 16S rRNA gene, or the fungal ITS2 region. For the bacterial 16S rRNA gene, amplicons were generated with 515F (5′-GTGYCAGCMGCCGCGGTAA-3′)^39^ and 806R (5′-GGACTACNVGGGTWTCTAAT-3′)^40^, while the fungal ITS2 region was amplified with primers ITS3 (5′-GCATCGATGAAGAACGCAGC-3′) and ITS4 (5′-TCCTCCGCTTATTGATATGC-3′)^41^. The pool of barcoded amplicons was sequenced at W.M. Keck Center for Comparative and Functional Genomics at the University of Illinois at Urbana-Champaign (Urbana, IL, USA) on Illumina MiSeq platform with a 2 × 250 bp read configuration (Illumina, San Diego, CA, USA). Each sequence was assigned to its original sample according to the barcode. After demultiplexing, the forward and reverse read of each paired-end sequence were merged using software FLASH (Fast Length Adjustment of Short reads)^42^. Filtered sequences were clustered into operational taxonomic units (OTUs) using USEARCH (v. 8.1.1861)^43^. USEARCH was used to (1) de-replicate sequences and remove singletons, and (2) remove chimeras contained in the sequences using GOLD as a reference database^44^ (http://drive5.com/uchime/uchime_download.html); (3) form clusters of 97% identity sequences and represent each OTU by consensus sequences (representative sequences). The cluster file was converted into an OTU table using a customized script derived from QIIME^45^. We used these consensus sequences as representative sequences in each OTU and the taxonomic attribution of filtered sequences was assigned in QIIME with uclust using 97% similarity score and 51% consensus against the default Greengenes database for 16S rRNA sequences, or a customized version of the UNITE database (http://unite.ut.ee) for fungal ITS2 sequences. Fewer than 1% of sequences across all samples received no taxonomic assignments and were removed from further analysis. The OTU table was subsampled to compensate for differences in total reads sequenced between samples. The OTU table was rarefied to equal abundance (9418 16S rRNA gene reads/sample, 1625 fungal ITS reads/sample) for further analyses. Samples producing fewer reads were eliminated from analysis. For this work, the UNITE database was customized by the addition of the *Ophidiomyces ophiodiicola* fungal ITS sequences. Mitochondrial, chloroplast, and protist sequences were removed from the OTU table. Bacterial 16S rRNA and fungal ITS2 DNA sequences are archived in Genbank, associated with BioProject accession number PRJNA478040.

### Statistical Analyses

Prevalence of skin lesions and *Ophidiomyces* as determined by qPCR was tabulated separately. Associations between categorical variables (presence of skin lesions, SFD presence, sex, location) were compared using a Pearson’s chi-squared test. Microbial sequence data were analyzed using multivariate correlational and ordination methods in the R statistical environment (R core Team, 2014), using the vegan R package (version 2.0–10)^46^. The effect size and statistical significance of samples classifications (SFD status of samples or individuals, year, field site, and sex) on bacterial and fungal community composition were determined using Permutational Multivariate Analysis of Variance (PERMANOVA, function adonis)^47^. Differences in community structure (16 S rRNA gene sequences, or fungal ITS2 sequences) were visualized with non-metric multidimensional scaling (NMDS) carried out on Bray-Curtis similarity indices calculated for each pairwise comparison of samples. Correlation of *Ophidiomyces* abundance with samples in ordinations was carried out with the function envfit and plotted on the NMDS plots. Analysis of similarity (ANOSIM; function anosim) was carried out to determine if samples within each classification were more similar to each other than to other samples^48^.

The phyloseq R package was used to summarize alpha and beta diversity of the bacterial and fungal DNA sequence datasets and to compare relative abundance of taxa^49^. Taxa that differed significantly between SFD-positive and -negative individuals were identified using the DeSeq2 package in R using the default parameters^50^. Alpha = 0.01 was used for determination of significantly different bacterial OTUs, and alpha = 0.05 was used to determine significantly different fungal OTUs.

## Electronic supplementary material

Supplementary Figures

## Data Availability

The datasets generated during and/or analyzed during the current study are available from the corresponding author on reasonable request.
