# Supplementary material for: Snake fungal disease alters skin bacterial and fungal diversity in an endangered rattlesnake
Source: Sci Rep. 2018 Aug 14;8:12147. doi: 10.1038/s41598-018-30709-x (PMC6092386; doi:10.1038/s41598-018-30709-x)
Supplement: Supplementary file 1 — Supplementary Figures [file 41598_2018_30709_MOESM1_ESM.pdf]

Snake fungal disease alters skin bacterial and fungal diversity in an endangered rattlesnake

Matthew C. Allender[1,2]*, Sarah Baker[1,2], Megan Britton[1], Angela D. Kent[3]

[1] Wildlife Epidemiology Lab, Department of Veterinary Clinical Medicine, College of Veterinary Medicine, University of Illinois Urbana-Champaign, Urbana, IL USA

[2] Illinois Natural History Survey, Prairie Research Institute, University of Illinois Urbana-Champaign, Champaign, IL USA

[3] Department of Natural Resources and Environmental Sciences, University of Illinois Urbana-Champaign, Champaign, IL USA

Supplemental figures

Figure S1: Nonmetric multidimensional scaling analysis of bacterial (A) and fungal assemblages (B) based on Illumina MiSeq sequencing of bacterial 16S rRNA genes or fungal ITS2, respectively. Each point represents a sample, and the distance between samples represents the Bray-Curtis dissimilarity values calculated among samples, coded by *Ophidiomyces* detection in the sample and year of sample collection.

Figure S2. Nonmetric multidimensional scaling analysis of bacterial (A) and fungal assemblages (B) based on Illumina MiSeq sequencing of bacterial 16S rRNA genes or fungal ITS2, respectively. Each point represents a sample, and the distance between samples represents the Bray-Curtis dissimilarity values calculated among samples, coded by *Ophidiomyces* detection in the sample and field location.

Figure S3. Nonmetric multidimensional scaling analysis of bacterial (A) and fungal assemblages (B) based on Illumina MiSeq sequencing of bacterial 16S rRNA genes or fungal ITS2, respectively. Each point represents a sample, and the distance between samples represents the Bray-Curtis dissimilarity values calculated among samples, coded by *Ophidiomyces* detection in the sample and body location.

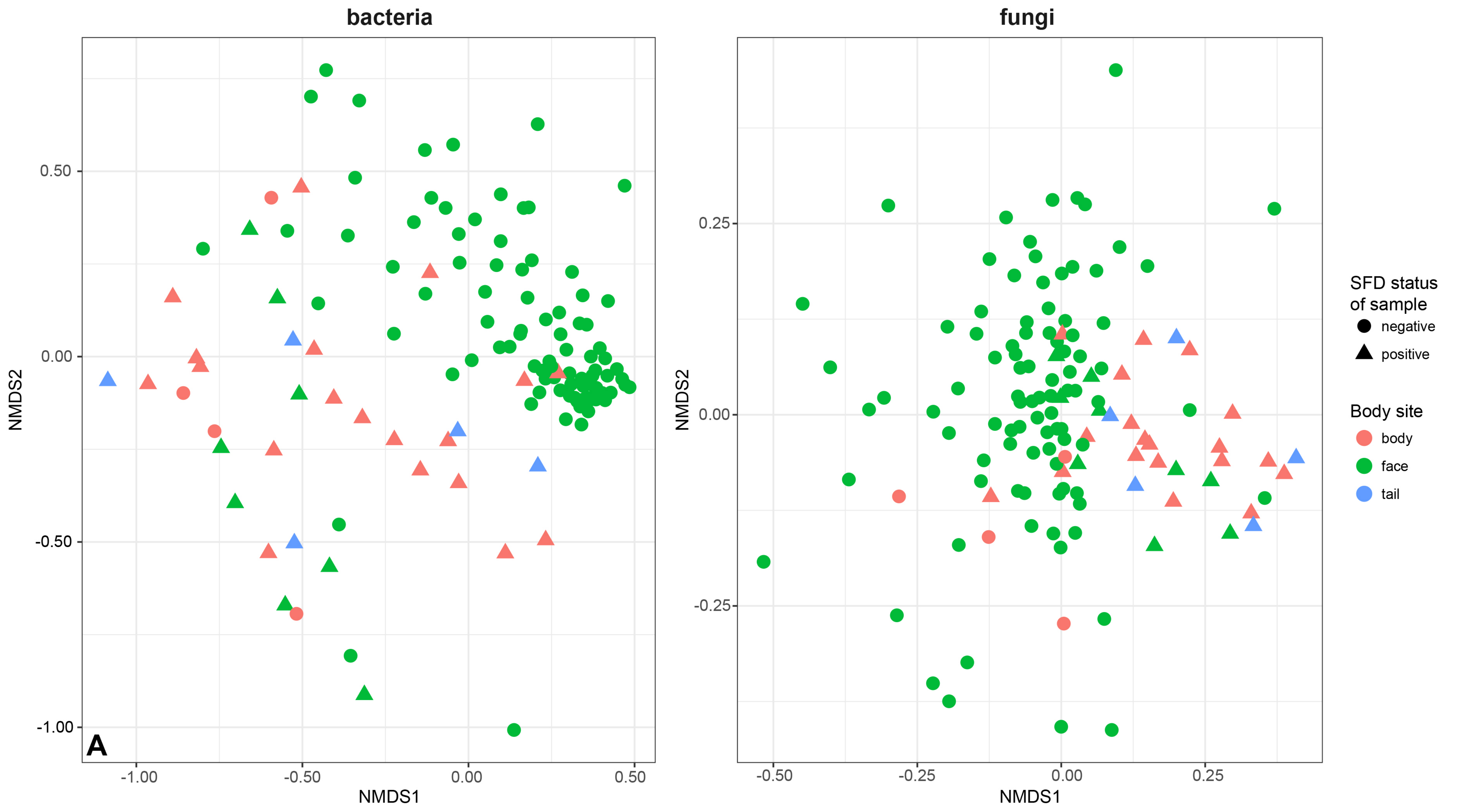

bacteria
fungi
NMDS1
NMDS2
0.50
0.00
-0.50
-1.00
-0.50
0.00
0.50
0.25
-0.25
A
SFD status
of sample
negative
positive
Body site
body
face
tail

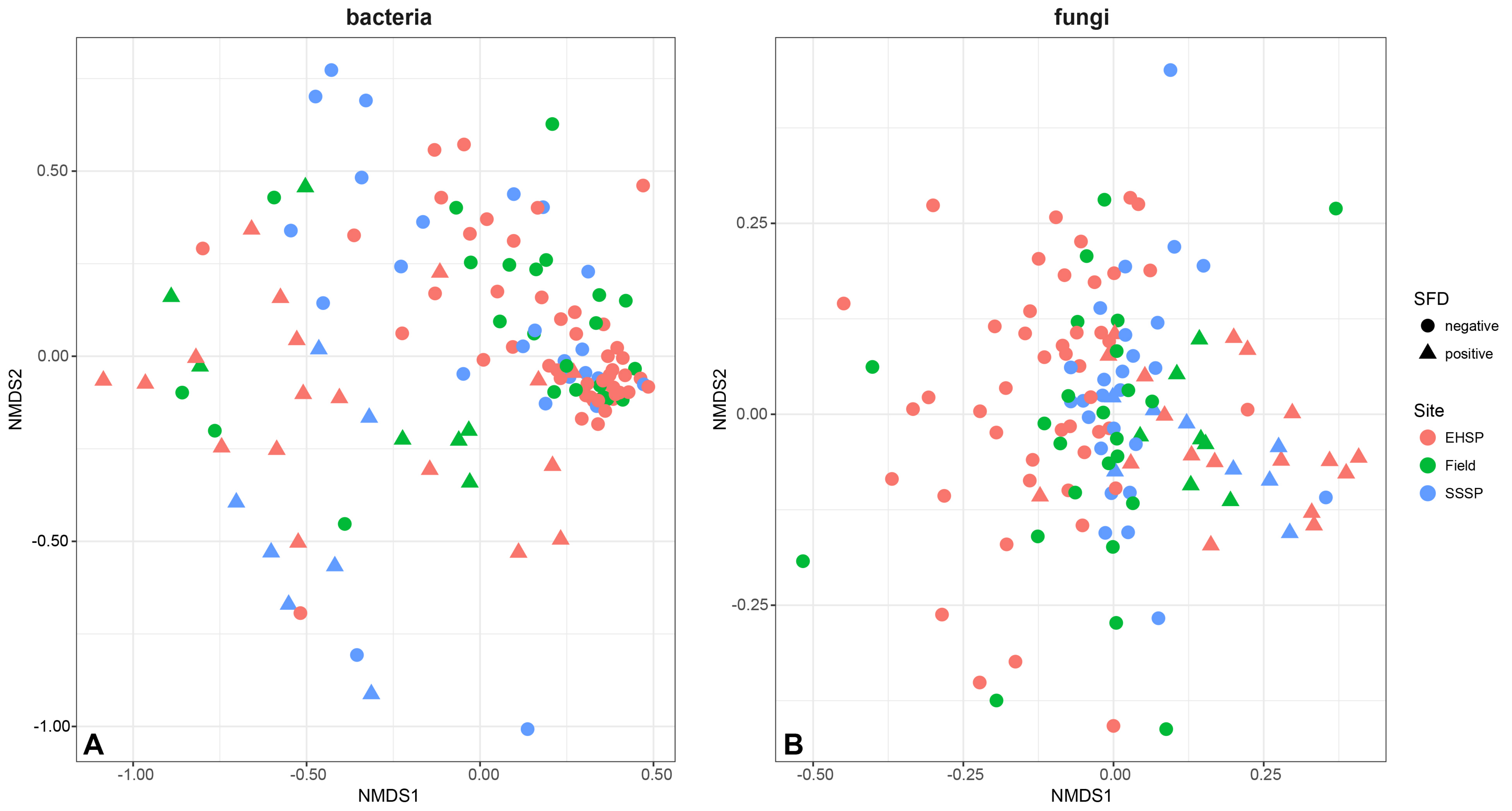
bacteria
fungi
NMDS1
NMDS2
0.50
0.00
-0.50
-1.00
-0.50
0.00
0.50
0.25
0.00
-0.25
-0.50
-0.25
0.00
0.25
A
B
SFD
negative
positive
Site
EHSP
Field
SSSP

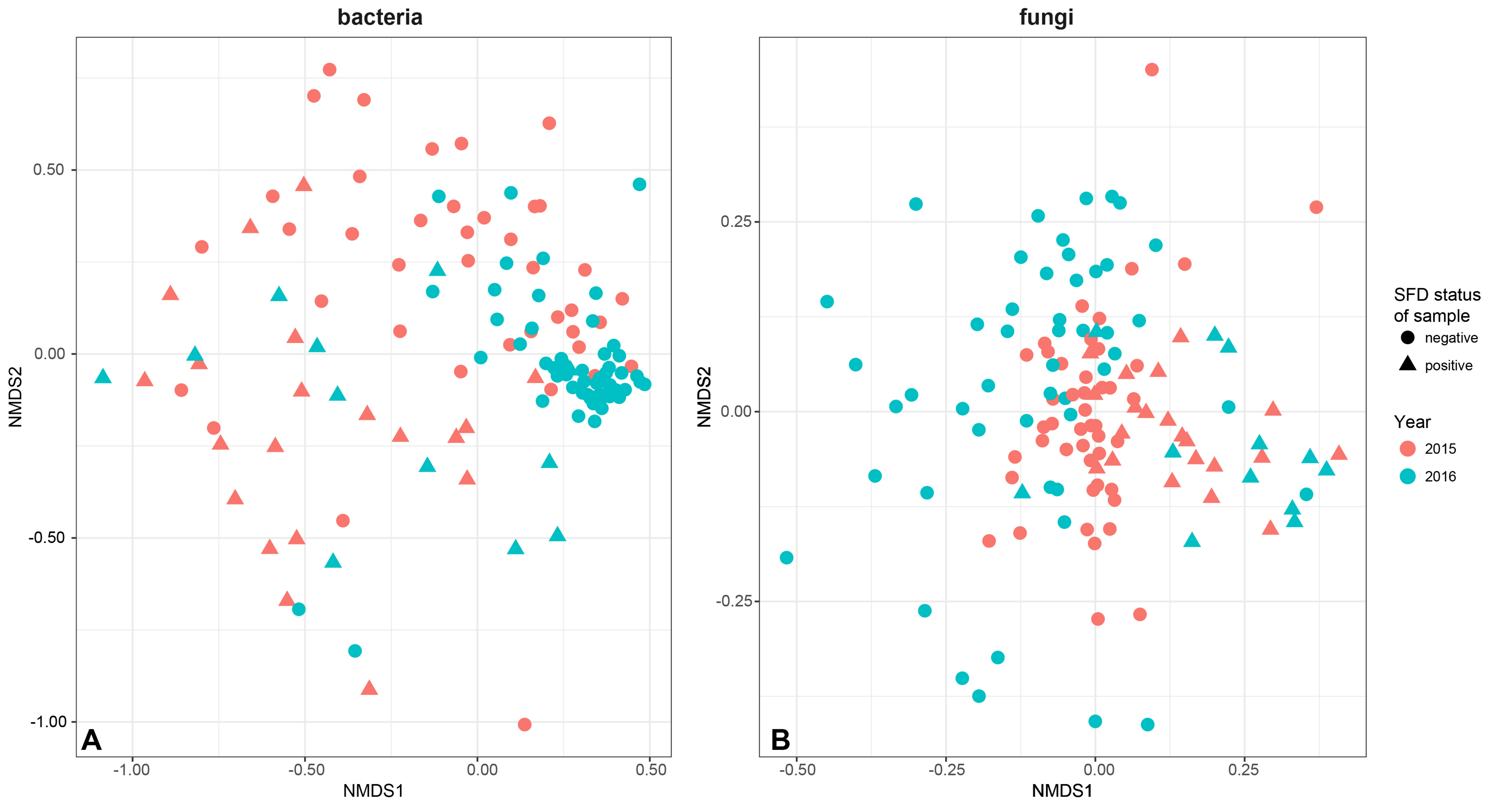
bacteria
fungi
NMDS1
NMDS2
A
B
-1.00
-0.50
0.00
0.50
-0.25
0.25
SFD status of sample
negative
positive
Year
2015
2016
